# Fighting Antibiotic Resistance in Hospital-Acquired Infections: Current State and Emerging Technologies in Disease Prevention, Diagnostics and Therapy

**DOI:** 10.3389/fmicb.2021.707330

**Published:** 2021-07-21

**Authors:** Ekaterina Avershina, Valeria Shapovalova, German Shipulin

**Affiliations:** ^1^Department of Biotechnology, Inland Norway University of Applied Sciences, Hamar, Norway; ^2^Laboratory or Postgenomic Technologies, Izmerov Research Institute of Occupational Health, Moscow, Russia; ^3^Federal State Budgetary Institution “Centre for Strategic Planning and Management of Biomedical Health Risks” of the Federal Medical Biological Agency, Centre for Strategic Planning of FMBA of Russia, Moscow, Russia

**Keywords:** antimicrobial resistance, ESKAPE pathogens, fast diagnostics, therapeutics, disease prevention

## Abstract

Rising antibiotic resistance is a global threat that is projected to cause more deaths than all cancers combined by 2050. In this review, we set to summarize the current state of antibiotic resistance, and to give an overview of the emerging technologies aimed to escape the pre-antibiotic era recurrence. We conducted a comprehensive literature survey of >150 original research and review articles indexed in the Web of Science using “antimicrobial resistance,” “diagnostics,” “therapeutics,” “disinfection,” “nosocomial infections,” “ESKAPE pathogens” as key words. We discuss the impact of nosocomial infections on the spread of multi-drug resistant bacteria, give an overview over existing and developing strategies for faster diagnostics of infectious diseases, review current and novel approaches in therapy of infectious diseases, and finally discuss strategies for hospital disinfection to prevent MDR bacteria spread.

## Introduction

Antimicrobial resistance (AMR) is an evolutionary response of bacteria, viruses, and fungi to withstand antimicrobial drugs introduced into their environment. In this systematic review we will focus on the resistance in bacteria only and the reader is advised to go through other reviews on viral and fungal resistance (Srinivasan et al., 2014; Fuentefria et al., 2018; Poole and James, 2018; Lampejo, 2020).

**FIGURE 1:**
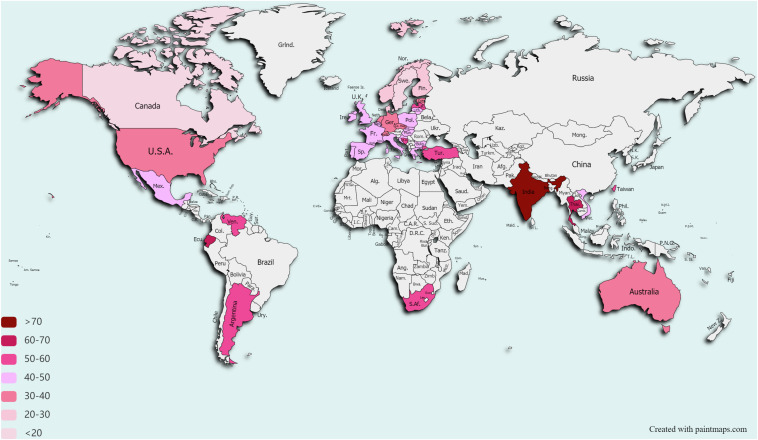
Drug Resistance Index worldwide. Only countries that reported data on antibiotic use for ≥5 pathogens and for ≥15 pathogen–antibiotic combinations for at least 1 year between 2012 and 2015 are depicted. Data taken from Klein et al. (2019). Data on Taiwan are taken from Resistance Map (https://resistancemap.cddep.org).

In 2020, the COVID-19 pandemic has clearly demonstrated how fragile our world is and how infections do not respect borders. However, we have long been living in a silent pandemic of AMR (Jasovsky et al., 2016). Each year, infections caused by resistant bacteria cause 68,000 deaths in the EU/EEA and the United States combined (Cassini et al., 2019; CDC, 2019), and are contributing to US €55 billion economic loss in the United States and to €1.6 billion in the EU/EEA annually (Ahmad and Khan, 2019). Although the AMR problem is not evenly distributed across the globe (Figure 1; Klein et al., 2019), there is no single country on Earth that can safely state that it will not be affected by the AMR spread. The first warning on a potential catastrophe came from Fleming (1945), in his speech on accepting the Nobel prize for discovery of penicillin, the first industrially produced antibiotic. Although he did not foresee the global spread of antibiotic resistance, he was the first one to recognize the danger of the resistance for everyone who relies on these drugs. In 2019, the Center for Disease Control and Prevention (CDC) released a report stating that we have entered the post-antibiotic era (CDC, 2019).

Antibiotics are classified by their chemical structure and have different modes of action, including cell wall synthesis inhibition (e.g., beta-lactams, fosfomycin, and vancomycin), DNA replication inhibition (e.g., fluoroquinolones), protein synthesis inhibition (e.g., tetracyclines and aminoglycosides), metabolic pathways inhibition (e.g., trimethoprim and sulfonamides) (Ma et al., 2020). The 1950 and 1960-s are a “Golden Era” of antibiotics (Lyddiard et al., 2016). More than half of currently used antibiotic classes were discovered at that time and majority of antibiotic scaffolds were derived from bacteria and fungi. *Actinomycetes* spp., for example, gave rise to 14 different drug classes, including carbapenems, aminoglycosides, glycopeptides, and lipopeptides (Hutchings et al., 2019). By the end of 1960s, novel scaffolds became more and more difficult to find and antibiotic discovery stalled. Cyclic lipopeptides were the last novel naturally derived antibiotic class that was discovered in 1987 and introduced into clinics in 2003 (daptomycin) (Eisenstein et al., 2010; Miller et al., 2016). Since that time, none of novel natural-product antibiotic scaffolds were discovered, although new antibiotics within existing classes are being constantly designed and developed. For example, in 2019 the FDA approved clinical use of Xenleta (Nabriva Therapeutics, Ireland), a drug from a pleuromutilin class, that was first discovered back in 1951 from a fungal source (Novak and Shlaes, 2010).

Some bacteria are intrinsically resistant toward some antibiotics due to their cell wall structure, activity of efflux pumps or presence of porins (Eichenberger and Thaden, 2019). In this case, all the strains within the species are insensitive to a given antibiotic. Acquired resistance, on the other hand, appears when some of the strains within the species become resistant toward the antibiotic they were previously susceptible to. Mechanisms include either mutations in existing genes, for example in intracellular targets (Musser James et al., 2020) or core metabolic genes (Lopatkin et al., 2021) or acquisition of new antibiotic resistance genes (ARGs) through horizontal gene transfer (HGT) (Eichenberger and Thaden, 2019). The latter enables intra- and inter-species transmission of ARGs and is responsible for an AMR pandemic (von Wintersdorff et al., 2016; Sun et al., 2019). Although ARGs existed long before antibiotics became discovered and put into a wide use (Hall and Barlow, 2004; Allen et al., 2010), our irresponsible use of antibiotics in animal husbandry, overuse of antibiotics in health care, improper treatment of wastewaters and poor sanitation in low and middle income countries has led to wide spread of resistant bacteria across the globe (Prestinaci et al., 2015; Holmes et al., 2016; Ma et al., 2020). To date, the most worrisome global spread of ARGs is the plasmid-mediated spread of carbapenemases (KPC, NDM, VIM, OXA-48, and OXA-51); colistin-resistance genes (*mcr*) in *Enterobacteriaceae*, *Acinetobacter baumannii*, and *Pseudomonas aeruginosa*; vancomycin resistance gene (*vanA*) in *Enterococci* and *Staphylococcus aureus*; and methicillin resistance gene (*mecA*) in *S*. *aureus* (de Niederhausern et al., 2011; Poirel et al., 2014; Lerner et al., 2015; Wang R. et al., 2018; Chaalal et al., 2020; Hishinuma et al., 2020; Snitser et al., 2020).

Health-care-associated infections (HAI) comprise infections that are acquired from hospitals or health care centers. They normally onset 48 h after hospitalization, but may occur even after discharge of patients (Revelas, 2012; Cassini et al., 2019). On average they occur in 7 and 10% of hospitalized patients in developing and developed countries, respectively (WHO, 2015). In Europe, around 3.2 million patients per year are affected by HAI (ECDC, 2013). The severity of infection and its incidence is directly correlated to the patients’ immunological status. Patients of burn units, intensive care units (ICUs), organ transplant receivers and neonates are the most affected groups. As such, a recent retrospective study from Serbia reported nearly every third patient admitted to ICU suffered at least one incident of HAI (Despotovic et al., 2020). HAIs are also responsible for three out of four lethal cases in neonates in Sub-Saharan Africa and South-East Asia (WHO, 2011). The most common HAI types are surgical site infections (2–5% incidence rate), catheter-related blood stream infections (12–25% incidence rate), catheter-related urinary tract infections (12% incidence rate) and ventilator-associated pneumonia (9–27% incidence rate) (Khan et al., 2017).

The World Health Organisation (WHO, 2017) stratified the most critical pathogens within HAI into three groups based on their global threat and urgency of action needed. The first and the second groups (urgent and high priority pathogens) include so-called ESKAPE pathogens (vancomycin-resistant *Enterococcus faecium* (VRE), methicillin-resistant, and vancomycin-resistant *S*. *aureus* (MRSA/VRSA), carbapenem-resistant and third-generation cephalosporin-resistant *Klebsiella pneumoniae*, *A*. *baumannii*, *P*. *aeruginosa*, and *Enterobacter* spp.). ESKAPE pathogens are associated with high morbidity and mortality HAIs due to their acquired resistance toward a large number of antibiotics, including last-resort antibiotics such as carbapenems and colistin (Santajit and Indrawattana, 2016; Ma et al., 2020). As such, a recent study from Greece reported that 90-days mortality risk was doubled in patients with HAI caused by carbapenem-resistant pathogens compared to patients without HAI (Kritsotakis et al., 2017). It is worth noting, though, that Greece is a country with highest consumption of antibiotics and with second largest disability-adjusted life years burden due to AMR in the EU (OECD, 2019).

Numerous reports are available on detecting resistant pathogens (including ESKAPE) in common areas in the hospitals (Li et al., 2015; Mirhoseini et al., 2016; Chng et al., 2020; Rodrigues et al., 2020). In a recent study from a tertiary-care hospital in Singapore (Chng et al., 2020), authors have collected swabs from both frequent contact- (bed rails, bedside lockers, and door knobs) and low contact areas (sink traps and aerators). They report a very worrisome spread and persistence of MDR organisms, some with novel and clinically dangerous ARG combinations. The authors also detected a plasmid containing both methicillin resistance gene *mecA* and genes for resistance toward disinfectants (*qacA* and *qacC*). Alarmingly, many of the MDR isolates have persisted in the hospital for nearly a decade, leading to opportunistic infections in hospital patients (Chng et al., 2020).

Antibiotic resistance is transmitted between animals and humans via the environment. For example, swine farming wastes can contaminate groundwater with ARGs and resistant bacteria, which can then colonize humans (Gao et al., 2020). Colistin (*mcr*) and carbapenem (NDM and VIM) resistance genes were detected in *Salmonella* isolates from food animals raising the danger of resistant bacteria infection through foodborne pathogens (Mthembu et al., 2021). NDM-1 gene was first detected in 2008 on a transferable plasmid in *K*. *pneumoniae* isolate from a patient who had been repatriated to Sweden from a New Delhi hospital (India) (Yong et al., 2009). Few years later it was detected in drinking and seepage water in New Delhi (Walsh et al., 2011) and in the river in Vietnam (Isozumi et al., 2012). Nowadays, NDM-1 and its variants are spread across the globe (Farhat and Khan, 2020), and were even detected in Arctic soil of remote region on Svalbard (Norway) with no agriculture or industry and <120 inhabitants, implicating possible spread via migrating birds (McCann et al., 2019). The One Health holistic approach is vital for mitigation of the AMR spread. One Health is a multi-sectoral concept recognizing interconnection between animal health, human health and the environment (Kim and Cha, 2021). It seeks for multidisciplinary effort to reduce dissemination of resistant bacteria worldwide through interventions in agriculture (and aquaculture), veterinary science, health sector, and sanitation. However, with multifactorial system in mind, human-to-human transmission still seemed to be the major contributor to the extended-spectrum beta-lactamase (ESBL) producing *E*. *coli* spread in the Netherlands community (Mughini-Gras et al., 2019). A recently published mathematical model for reduction of antibiotic resistant bacteria in humans also corroborates these findings (Booton et al., 2021). The model was based on the actual prevalence of ESBL-producing bacteria in Thailand and One Health drivers were weighted with regards to their sole or interactive impact on resistant bacteria dissemination in humans. Based on their model, reduction of human antibiotic use seems to be the most influential factor on the reduction of colonization of humans by resistant bacteria in a 20-years perspective.

Apart from educational, regulatory and political initiatives (Ahmad and Khan, 2019; Cole et al., 2019; Hernando-Amado et al., 2019; Ardal et al., 2020), there are three pillars to reduce the human impact on AMR spread. We need to develop and implement improved disinfection and hygiene routines in order to curb resistant pathogens persistence. We also need to develop new, fast and precise tools for infection diagnostics. Last, but not least, we have to search for new therapeutic strategies to combat resistant bacterial infections.

## Prevention of Infection

Majority of infections caused by MDR bacteria are of nosocomial origin (Matta et al., 2018), and these MDR pathogens can reside in hospitals for years (Chng et al., 2020). Several reports have demonstrated that a risk of acquiring HAI increases if a previous patient in a room had HAI (Mitchell et al., 2015). Unfortunately, hospitals disinfection is often only scarcely touched upon when discussing AMR spread issue, and current cleaning routines have remained largely unrevised for the last 25 years (Peters et al., 2018). The main global change in Infection Prevention and Control routines was the introduction of alcohol-based handrubs by the WHO in 2005 (Pittet and Donaldson, 2005), the routine which is followed in over 180 countries worldwide (Peters et al., 2018). However, based on a recent joint report of healthcare facilities published by the WHO and UNICEF, every third facility globally lacks adequate hand cleaning at points of care, and two out of three facilities in least developed countries do not have adequate waste management service (WHO, 2020). Hospital wastewaters contain high levels of resistant bacteria, ARGs and mobile genetic elements (Wang Q. et al., 2018) and strongly contribute to their dissemination in the environment even after treatment (Buelow et al., 2020). A recent study from Germany indicated, that effluents from hospital wastewater treatment plants may contain up to 70% higher daily discharge of NDM-1, *mcr-1*, *vanA*, and *mecA* genes compared to communal or food production wastewater treatment plants although median daily discharge values did not differ (Alexander et al., 2020).

### Hospital Surfaces and Wastewater Treatment

Commonly used chemical disinfectants for cleaning hospital surfaces and waste waters include chlorine and chlorine-based products, hydrogen peroxide, alcohol (ethanol, isopropanol), quaternary ammonium compounds, and formaldehyde (Peters et al., 2018). Hydrogen peroxide vapor (HPV) used for surface disinfection, is sometimes combined with silver ions (Totaro et al., 2020). In a field study, 30% HPV-Ag+ treatment of hospital rooms eliminated MRSA from the hospital surfaces with no regrowth after 2 weeks (Bartels et al., 2008). Hydrogen peroxide is also most effective for eliminating resistant *A*. *baumannii* compared to sodium hypochlorite and chlorine dioxide (Biswas et al., 2018). H_2_O_2_ is utilized in so-called electroperoxone (E-peroxone) process for waste water treatment (Wang Y. et al., 2018). In this process, H_2_O_2_ generated by electrical process, reacts with ozone and reduces ARGs count (Zheng et al., 2018). Unfortunately, chemical agents can promote HGT in pathogens and thus contribute to AMR spread (Alotaibi et al., 2017; Lu and Guo, 2021). For example, hospital *K*. *pneumoniae* strains harboring numerous ARGs, can become tolerant to chlorine, and can then escape into environment through hospital wastewaters (Popa et al., 2020).

Physical processes are more difficult to develop resistance toward. UV-C light (wave length 200–280 nm) causes multiple DNA lesions which are difficult to repair (Clancy, 2008). It is an effective disinfection strategy for hospital surfaces (Rastogi et al., 2010). For example, mobile Hyper Light P3 disinfection robot utilizing UV-C is effective for killing MDR pathogens in hospitals, albeit at the distance of 1 m only, with reduced effect at 2 or 3 m (Yang et al., 2019). Efficacy of UV treatment is dependent on the GC-content of bacteria, with GC-rich bacteria being more resilient to UV light treatment (Pullerits et al., 2020). As such, >20 min of UV light treatment is required to reach 3-log reduction of *Mycobacterium tuberculosis* (GC ≥ 65%) compared to <10 min for MRSA (GC ≈ 33%) or extended spectrum beta-lactamase (ESBL) producing *E*. *coli* (GC ≈ 50%) (Szeto et al., 2020).

To avoid bacterial contamination, medical tools are often coated with silver nanoparticles (NPs) (Deshmukh et al., 2019). ZnO NPs were suggested for cleaning hospital surfaces contaminated with resistant *P*. *aeruginosa* (Omrani and Fataei, 2018). Ag–NPs–TiO_2_-embedded filters installed in ventilation systems of hospital ward demonstrated 88% bacterial removal within 30 min (Chen et al., 2019). Ag–NPs in combination with TiO_2_ films may also serve as a photocatalyst for photoinactivation of bacteria in visible indoor light (Dunnill et al., 2011). MoS_2_/α-NiMoO4 nanostructure developed Ray et al. (2020), also disrupts bacterial cell at visible light. It has been shown to inactivate *S*. *aureus* and was proposed to be used to decontaminate MDR-containing waste waters. Combination of NPs with bacteriophages can also be successfully implemented for waste water treatment in hospitals (Talan and Tyagi, 2020). It is very important to be careful with NPs concentration used for waste water treatment. It has been shown that ZnO NPs at low concentrations can enhance horizontal transfer of ARGs between bacteria (Wang X. et al., 2018). However, it has been also shown that despite enhancement of HGT mechanism, metal NPs ultimately kill pathogens and attenuate ARGs (Su et al., 2019).

### Medical Devices

Sterility of medical devices or implants is crucially important to prevent patient’s infection. Plants and insects utilize a variety of nanostructured antibacterial surfaces for protection against pathogenic bacteria. For example, cicada wings are covered with 200 nm long nanoneedles, and gecko’s skin is shielded by hair-like curved nanostructures (Tripathy et al., 2017). These surfaces physically disrupt bacteria cell wall killing the pathogen within minutes after the initial contact (Ivanova et al., 2012; Watson et al., 2015). Majority of these surfaces are active only against gram negative bacteria due to their thin cell wall. However, inspired by naturally occurring bactericidal nanosurfaces, scientists created a plethora of artificial nanostructured coating materials that are lethal to gram positive bacteria as well. These materials include nanocone surfaces, nanowires, nanograss, nanospikes, nanopillars, and nanorings, etc. (Hazell et al., 2018; Gonzalez-Fernandez et al., 2020; Gudz et al., 2020; Jenkins et al., 2020). For example, black silicon (nanograss) was created by mimicking cicada wings nanoneedles, which are lethal not only to gram-negative *P*. *aeruginosa*, but also to gram-positive *S*. *aureus* and *B*. *subtilis* (Ivanova et al., 2013). Additionally, recently a nanostructure with a combination of antiviral and antibacterial properties was reported (Hasan et al., 2020).

There are several considerations to be resolved before nanostructures can be put to a wide use for medical devices. Many of these structures were shown to disrupt not only bacterial, but also mammalian cells (Shalek et al., 2010; Pham et al., 2014). However, recently investigators report little to no adverse effect on mammalian cells (CLSI, 2019; Wandiyanto et al., 2019; Saini et al., 2020). Large scale fabrication of these materials is also costly, although production of nanospikes, nanorods, nanowires, and nanoneedles can be achieved at low cost (Tripathy et al., 2017; EUCAST, 2021).

### Healthcare Workers Clothing and Personal Belongings

Even when perfectly following hand hygiene, resistant bacteria reside on workers’ clothing and hospital textiles (Lena et al., 2021). These bacteria can further contaminate laundry facilities, washing machines, and cause pathogen outbreaks (Michael et al., 2017). For example, and outbreak of ESBL-producing *K*. *pneumoniae* in a 40-bed rehabilitation center in the Netherlands was traced back to a contaminated washing machine. The outbreak was contained after taking the machine out of service and reinforcing its use protocols (Boonstra et al., 2020). For the efficient pathogen removal from textiles and clothing, water temperature, bleach use and mode of drying are essential (Tano and Melhus, 2014; Bockmuhl, 2017). As such, laundering at 60°C is recommended for complete removal of bacteria (Riley et al., 2017). In recent years, in order to reduce energy consumption, wash temperatures have been lowered to below 60°C, which may compromise the efficacy of pathogen removal and lead to clothing re-contamination (Riley et al., 2017; Bockmuhl et al., 2019). Moreover, in the United Kingdom, for example, healthcare workers uniforms are washed at home to reduce National Health Service costs, and many workers do not follow the washing recommendations (Riley et al., 2015).

Healthcare workers’ mobile phones can also be a source of transmission of resistant bacteria to the community (Debnath et al., 2018; Mushabati et al., 2021). In EU, this problem seems to be less striking with fewer resistant bacteria detected on the phones (Galazzi et al., 2019; Missri et al., 2019). However, routines for mobile phone disinfections should be implemented and strictly followed worldwide.

### Perception of the Cleaning Service

No matter how good disinfectants and strategies we use, the key is to use them properly, at a right dosage, time, for the right bug and equipment, and strictly following the protocols. However, cleaning service is perceived as a low qualified job and there is a high turn-around among the personnel in hospitals. In addition to low rang, many workers in cleaning service in high-income countries are immigrants, often with very limited skills in local language (Peters et al., 2018). This makes it difficult to convey an importance of such a “mundane” but yet crucially important task to the personnel. Therefore, we also need to break the perception of cleaning in hospitals as a dull and low-quality job and to use well trained and certified cleaners for such critically important areas as hospital is.

## Rapid Diagnostics Technology

Classical phenotypic antibiotic susceptibility testing (AST) refers to broth microdilution (BMD) or diffusion (Khan et al., 2019). BMD is regarded as gold standard MIC breakpoint determination both by the Clinical and Laboratory Standards Institute (2019) and the European Committee on Antimicrobial Susceptibility Testing (2021). Number of automated systems for AST testing have been approved for commercialization by the United States Food and Drug Agency (FDA), like Vitek2 (BioMérieux, France), Microscan (BeckmanCoulter, United States), Phoenix (BD diagnostics, United States), and Sensititre (ThermoFisher, United States).

Although quite precise, these systems require long time for MIC determination and are limited to a certain species/drug panel. For instance, common time for obtaining AST profile of an infectious agent in bloodstream infections may be as long as 1 week after the initial diagnosis has been made (Briggs et al., 2021). This waiting time would be lethal for a patient and therefore doctors start with broad-spectrum antibiotics until the AST results come in Kumar et al. (2009). Ideally, broad-spectrum antibiotics should be rapidly de-escalated toward optimal antibiotic to minimize their use and thus to reduce the risk of resistance development. Rapid diagnostics tools enable AST inference within hours, providing an opportunity for fast shift toward optimal antibiotics use. In a retrospective study from a tertiary care children’s hospital, Reuter et al. (2019) reported a significant reduction in duration of suboptimal antibiotic treatment when rapid AST was performed. Recently EUCAST has developed a rapid antibiotic susceptibility testing (RAST) method directly from positive blood culture bottles. The method is based on disk diffusion with shortened incubation times of 4–8 h and was extensively validated in clinical labs across 55 European countries (Åkerlund et al., 2020). A plethora of rapid diagnostic tools based on molecular methods such as fluorescence microscopy, proteins detection, hybridization, nucleic acid amplification technologies, and immunodetection are being currently developed [reviewed in detail in Vasala et al. (2020) and van Belkum et al. (2020)]. Here we will give an overview on gold standard rapid diagnostics, genotypic (detect potential antibiotic resistance), and phenotypic (detect actual antibiotic resistance) point-of-care tools approved by FDA and touch on emerging technologies for future rapid diagnostics (Table 1).

**TABLE 1:** Overview of alternatives to classical antibiotic susceptibility testing (AST) discussed in this review.

Technology	Instrumentation	FDA-approved assays	Targets	Resistance to	Preliminary culture	Culture isolation	Genotypic/phenotypic	Pros	Cons
MALDI-TOF MS	MALDI Biotyper (Bruker, Germany)	IVD-CE	>300 bacteria, 10 yeasts	-	Yes	Yes	Phenotype	Save at least 24 h compared to classical AST	Costly (operation costs ∼€200 000 per year)
		MBT STAR-Cepha assay	*Enterobacteriaceae*	Cephalosporins					
		MBT STAR-Carba assay	–	Carbapenemases					
	VITEK MS (BioMerieux, France)	V3/KB V3.2.0	>1,300 bacteria, yeast, and moulds	–					
Multiplex PCR	Xpert^®^ (Cepheid, United States)	MRSA/SA	*Staphylococcus aureus*	Methicillin	Yes	No	Genotype	Save 24–48 h compare to classical AST	Do not provide phenotypic AST; do not distinguish between expressed/non-expressed genes
		Xpert^®^ MTB/RIF	*Mycobacterim tuberculosis*	Rifampicin					
		Xpert Carba-R	–	Carbapenems					
		XPert^®^ *vanA*	VRE	Vancomycin					
	BioFire^®^ FilmArray^®^ (BioMérieux, France)	BCID2 (blood infections)	ESKAPE; *Salmonella*; *Serratia marcescens*; *Neisseria meningitis*; *B*. *fragilis*; *Listeria monocytogenes*; and *Streptoccoccus*	Methicillin (*mecA/C*); vancomycin (*vanA/B*); carbapenems (IMP, KPC, OXA-48-like, NDM, VIM); colistin (*mcr-1*); and CTX-M genes	Yes	No	Genotype	Syndromic approach; save at least 24 h compared to classical AST	Do not provide phenotypic AST; do not distinguish between expressed/non-expressed genes
		PN (pneumonia)	ESKAPE; *Serratia marcescens*; *Proteus* spp.; *Moraxella catarrhalis*; *Chlamydia pneumoniae*; *Legionella pneumophyla*; and *Mycoplasma pneumoniae*	Methicillin (*mecA/C*); carbapenems (IMP, KPC, OXA-48-like, NDM, VIM); and CTX-M genes	No				
	Unyvero (Curetis)	Invasive joint infection	ESKAPE; *Proteus* spp.; *Propionibacterium acnes*; *Finegoldia magna*; and *Bacteroides fragilis*	Methicillin (*mecA/C*); vancomycin (*vanA/B*); macrolide/lincosamide (*ermA/C*); aminoglycoside [*aac*(*6’*)*/aph*(*2”*); *aacA4*]; third gen cephalosporins (CTX-M); carbapenems (KPC; IMP; NDM; VIM; OXA-23; OXA-24/40; OXA-48; and OXA-58)	No	No	Genotype		
		Urinary tract infection	ESKAPE; *Proteus* spp.; *Citrobacter freundii/koseri*; *Providencia* spp.; *Prevotella* spp; CoNS; and *Corynebacterium urealyticum*	Methicillin (*mecA/C*); vancomycin (*vanA/B*); third gen cephalosporins (CTX-M); carbapenems (KPC; IMP; NDM; VIM; OXA-23; OXA-24; OXA-48); colistin (*mcr-1*); fluoroquinolones (*qnrB/S*); and sulfonamide (*sul1*)	No	No	Genotype		
		Intra abdominal infection	ESKAPE; *Proteus* spp.; *Citrobacter freundii/koseri*; *Bacteroides* spp.; *Prevotella* spp; CoNS; *Clostridioles difficile*; *C*. *Perfringens*; *Finegoldia magna*; and *Cutibacterium acnes*	Methicillin (*mecA/C*); vancomycin (*vanA/B*); aminoglycoside (*aacA4*); third gen cephalosporins (CTX-M); carbapenems (KPC; IMP; NDM; VIM; OXA-23; OXA-24; OXA-48; OXA-58); colistin (*mcr-1*); nitroimidazole (*nimA/B*); fluoroquinolones (*qnrA/B/S*); and tetracycline (*tetA*)	No	No	Genotype		
		Blood stream infection	ESKAPE; *Proteus* spp.; *Citrobacter freundii/koseri*; *Prevotella* spp; CoNS; *Listeria monocytogenes*; *Serratia marcescens*; *Stenotrophomonas* and *maltophilia*	Methicillin (*mecA/C*); vancomycin (*vanA/B*); macrolide/lincosamide (*ermA*); aminoglycoside [*aac*(*6’*)*/aph*(*2”*); *aacA4*]; third gen cephalosporins (CTX-M); carbapenems (KPC; IMP; NDM; VIM; OXA-23; OXA-24/40; OXA-48; OXA-58)	Yes	No	Genotype		
		Lower respiratory tract	ESKAPE; *Chlamidia pneumoniae*; *Citrobacter freundii*; *Haemophilus influenzae*; *Legionella pneumophila*; *Moraxella catarrhalis*; *Morganella morganii*; *Micoplasma pneumoniae*; *Proteus* spp.; *Serratia marcescens*; *Stenotrophomonas maltophilia*; and *Streptococcus pneumoniae*	Methicillin (*mecA*); penicillin (TEM); third gen cephalosporins (CTX-M); and carbapenems (KPC; IMP; NDM; VIM; OXA-23; OXA-24; OXA-48; and OXA-58)	No	No	Genotype		
Microarray	Verigene^®^ (Limunex, United States)	Bloodstream infection testing panel	ESKAPE; *Micrococcus* spp.; *Citrobacter* spp.; and *Proteus* spp.	Methicillin (*mecA/C*); vancomycin (*vanA/B*); carbapenems (IMP, KPC, OXA, NDM, VIM); CTX-M genes	Yes	No	Genotype	Syndromic and targeted approach; save at least 24 h compared to classical AST	Do not provide phenotypic AST; do not distinguish between expressed/non-expressed genes
FISH and morphokinetic cell analysis	Accelerate Pheno (Accelerated Diagnostics, United States)	Accelerate PhenoTest^®^ BC Kit (blood infections)	ESKAPE; *S*. *marcensens*; *Citrobacter* spp.; and *Proteus* spp.	Cefoxitin (=methicillin); vancomycin; third gen cephalosporins; carbapenems; and aminoglycosides	No	No	Phenotype	Provides ID & AST; saves up to 40 h compared to classical AST	Costly (∼€250 per sample)
Colorimetry	RAPIDEC (BioMerieux)	RAPIDEC Carba-NP	–	Carbapenems	Yes	Yes	Phenotype	Saves up to 24 h compared to classical AST	Cannot process clinical samples, need pure isolates
Immunochromatography	*K*-SeT (CorisBio, Belgium)	OXA-48 *K*-SeT	CPE	Carbapenems (OXA-48)	Yes	Yes	Phenotype		
		RESIST-3 O.K.N. *K*-SeT	CPE	Carbapenems (OXA-48; KPC; NDM)	Yes	Yes	Phenotype		
		RESIST-4 O.K.N.V. *K*-SeT	CPE	Carbapenems (OXA-48; KPC; NDM; and VIM)	Yes	Yes	Phenotype		
	NG-Test (NG Biotech, France)	NG-Test CARBA 5	CPE	Carbapenems (OXA-48; KPC; NDM; VIM; and IMP)	Yes	Yes (no for blood culture)	Phenotype		
		NG-Test CTX-M MULTI	ESBL-producing bacteria	CTX-M-15 group; CTX-M-2 group and CTX-M-14 group	Yes	Yes	Phenotype		
		NG-Test MCR-1	Colistin resistant bacteria	MCR-1	Yes	Yes	Phenotype		
Microfluidics/biosensors	BYG Carba (Bogaerts et al., 2017)	–	CPE	Carbapenems	Yes	Yes	Phenotype	Provides AST; saves up to 24 h compared to classical AST	Cannot process clinical samples, need pure isolates
	Microcolorimetry AST plate (Elavarasan et al., 2013)	–	*E*. *coli*; *Shigella*	Amikacin; gentamicin; kanamycin; and ampicillin	Yes	Yes	Phenotype	Provides AST; saves up to 18 h compared to classical AST; cost efficient (low reagent and sample volumes)	Cannot process clinical samples, need pure isolates
	Droplet microfluidic platform (Kang et al., 2019)	–	*S*. *aureus*; *E*. *faecalis*; *E*. *coli*; and *K*. *pneumoniae*	Oxacillin and tetracycline	Yes	Yes	Phenotype	Provides AST; saves up to 24 h compared to classical AST	Cannot process clinical samples, need pure isolates
WGS	Whole genome sequencing (Taxt et al., 2020) (can be used in combination with CRISPR/cas targeting system)	–	Any	Any	Yes	No	Genotype	Can detect bacterial ID within minutes after loading, resistance genes–within an hour; is independent of mutations, can detect taxonomy and any resistance gene; can be coupled with ML algorithms for prediction of phenotype	Does not distinguish between expressed and non-expressed genes

### Matrix-Assisted Laser Desorption/Ionization Mass Spectrometry MALDI-TOF MS

The gold standard rapid bacteria identification technology is matrix-assisted laser desorption/ionization mass spectrometry (MALDI-TOF MS) that identifies bacteria by comparing its protein profile to a reference library within minutes after the sample is loaded. The FDA-approved Bruker MALDI Biotyper (Bruker, Germany) uses a library of >300 clinically relevant bacteria and yeast (FDA, 2018; Florio et al., 2019). Bruker (Germany) also provides MBT STAR-Carba and MBT STAR-Cepha assays for detection of carbapenem and third gen cephalosporin resistance in *Enterobacteriaceae* (both assays), *Acinetobacter* spp. (MBT STAR-Carba only), and *Pseudomonas* spp. (MBT STAR-Carba only) within 1 h after loading.

The VITEK^®^ MS (BioMérieux, France) reference database contains protein spectra of >1,300 species of bacteria, yeast and moulds. When coupled with Vitek2 (BioMérieux, France) for AST, the system can seamlessly provide simultaneous ID and AST of the pathogen. They also provide a VITEK^®^ MS Blood Culture kit for identification of blood stream infection directly from blood culture samples, but per date this kit is for research use only.

Although these systems allow rapid and accurate bacterial identification and AST profile, MALDI-TOF MS has two major drawbacks. It requires isolated colonies from overnight culture, saving only around 24 h compared to conventional AST (Briggs et al., 2021). The incubation time, however, can be shortened down to 3–6 h incubation prior to MALDI-TOF MS (Cherkaoui et al., 2020). Secondly, a MALDI-TOF MS system may cost up to €200,000 per year (excluding operational costs) rendering it impossible for use in outpatient clinics or small hospitals (Vasala et al., 2020).

### Genotypic Point-of-Care Technologies

Several multiplexed panels are FDA-approved for point-of-care use. Cepheid^®^ provides targeted test panels for detection of MRSA (Xpert^®^ MRSA/SA), *Mycobacterium tuberculosis* and rifampicin resistance (Xpert^®^ MTB/RIF), vancomycin-resistant enterococci (XPert^®^ *vanA*), and for carbapenem resistance detection (Xpert Carba-R) among others. BioMérieux BioFire^®^ FilmArray^®^ provides panels for simultaneous identification of up to 43 targets (bacteria and resistance genes) using syndromic approach for pneumonia and bloodstream infections. Unyvero (Curetis) syndromic panels cover bacteria and ARGs detection in invasive joint infections, urinary tract infections, intra-abdominal infections, blood stream infections and lower respiratory tract infections. All of these panels are based on multiplex PCR and require 1–5 h load to end time. The Verigene^®^ system by Luminex Corporation is based on microarray technology and has both targeted and syndromic panels for detection of main pathogens and resistance genes within a matter of hours. Although providing fast and broad detection of pathogens with good concordance to conventional methods (Briggs et al., 2021), these panels require a preliminary culturing step of clinical samples, which can take up to 24 h. Moreover, same as other nucleic-acid based technologies, they do not provide phenotypic AST and do not distinguish between expressed and non-expressed genes. In case of polymicrobial infections, they also cannot provide a pathogen/gene relation. The other approach can be to use a proxy for resistance instead of direct ARGs search. It has been noted that integron detection in bacterial DNA is highly correlated with antibiotic resistance of *Enterobacteriaceae* pathogens in blood cultures from septic patients (Barraud et al., 2014), opening the possibility for a rapid inference of antibiotic resistance and correction of the antibiotic prescription prior to detailed AST.

There is a potential for development of a rapid diagnostic tool using Whole Genome Sequencing (WGS). WGS provides excessive amount of genome information without the need for multiplex targeting of certain genes, bringing the advantage of wide coverage of variety of pathogens with one set up. Since the approach is non-targeted, it is also less sensitive to mutations in genes. For example, the multiplex PCR-based Xpert^®^ MTB/RIF panel (Cepheid^®^, United States) does not detect RpoB Ile149Phe mutation and reports false negative results (Zhao Z. L. et al., 2020). The other advantage of WGS is that it is capable of capturing slow-growing and fastidious bacteria, which may be troublesome or impossible to detect by growth-based methods (Balloux et al., 2018). With real-time sequencing technologies as ONT sequencing (Oxford Nanopore Technologies, United Kingdom), the sequencing information can be received within minutes after the sample load and bacterial ID and ARGs can be detected on the flow (Taxt et al., 2020).

Same as other nucleic-acid based technologies, however, current WGS methodologies do not allow direct clinical sample sequencing and require preliminary culturing. To avoid this step, CRISPR/Cas9 based detection of AMR sequences was proposed by Quan et al. (2019). This highly multiplexed approach enables precise and cheap detection of all known ARGs from low abundance samples. Here, genomic DNA from a patient sample is digested with Cas9 endonuclease guided by a set of RNAs designed specifically from target ARG sequences. Restricted fragments are then ligated to adaptors, enriched by PCR and sequenced. Although this approach was tested only on a handful of clinical samples, it exhibited promising results with extraordinary sensitive ARG detection surpassing direct NGS by 5,000-fold (Quan et al., 2019).

Antibiotic resistance gene detection does not always correlate to phenotypic AST profile of the pathogen, especially when changes in influx/efflux transport systems may render antibiotic inefficient due to its inability to accumulate in the cell (Masi et al., 2017). Here, various machine-learning (ML) algorithms may be implemented for genotype-to-phenotype correlations. Although these algorithms have not been introduced to a clinical routine per date, there are number of publications demonstrating the power of ML to predict susceptibility and MIC based on genomic features for beta-lactams, aminoglycosides, polymyxins, and other antibiotic classes (Nguyen et al., 2018; Kim et al., 2020; Macesic et al., 2020; Avershina et al., 2021). However, these models are species and drug specific and are dependent on the resistance mechanisms represented in a training set, limiting their use with isolates that possess other mechanisms/genomic signatures (Avershina et al., 2021).

### Phenotypic Point-of-Care Technologies

AcceleratePheno^TM^ (Accelerated Diagnostics, United States) is the only FDA-approved point-of-care rapid diagnostic platform that enables simultaneous identification and MIC-based AST profile of a pathogen directly from a clinical sample. For the moment, it covers 14 common infectious agents in bloodstream infections, including ESKAPE pathogens and two *Candida* species, and performs MIC testing to cefoxitin (a surrogate for methicillin resistance detection), vancomycin, third generation cephalosporins, carbapenems, and aminoglycosides. Pathogen identification is performed using fluorescence *in situ* hybridization and phenotypic AST is performed using morphokinetic cell analysis of microbial cells in an antibiotic-supplemented Mueller-Hinton media (Charnot-Katsikas et al., 2018). AcceleratePheno^TM^ allows up to 27 h faster bacterial ID and 40 h faster AST compared to conventional culture-based identification and AST (Marschal et al., 2017). However, this technology is yet costly (∼€250 per sample) for use in point-of-care centers (Vasala et al., 2020).

Colorimetric assays allow rapid identification of antibiotic resistant bacteria by changing the color of the medium on the change of pH resulting from the activity of resistance genes products. For example, RAPIDEC^®^ Carba-NP (BioMerieux, France) detects carbapenemase activity in ESKAPE pathogens with ∼95% sensitivity and specificity within 30 min–2 h after loading (Poirel et al., 2015; Mancini et al., 2017). However, colorimetric assays require at least overnight culture and can’t be used directly on the clinical sample.

Microfluidics holds a big promise for rapid AST technologies. These technologies operate with micro volumes and are thus portable and allow for multiplexing and cost-effectiveness. Resazurin dye can be used in microcolorimetry for AST when added to an antibiotic-supplemented medium. In presence of viable bacteria, resaruzin will be reduced and its color changed from blue to pink and leuco, an approach tested on *E*. *coli* and *Shigella* by Elavarasan et al. (2013). Kang et al. (2019) has reported an image-based parallelized droplet microfluidic platform capable of screening four bug-drug combinations simultaneously requiring 30 min–2 h for AST. Microfluidics is vastly used in various biosensors, analytical devices that detect a biological reaction by converting a chemical response into an electrical signal (Mehrotra, 2016). For example, change in pH caused by degradation of carbapenems by carbapenem-resistant *Enterobacteriaceae* (CPE) laid foundation for the development of the BYG Carba biosensor capable of detecting CPE within 30 min (Bogaerts et al., 2017). Immunochromatographic assays use monoclonal antibodies specific to resistance enzymes. OXA-48 *K*-SeT (Coris BioConcept, Belgium) was the first test developed for *in vitro* identification of OXA-48-like carbapenemases in bacterial cultures. Later, assays for additional detection of NDM and KPC (RESIST-3 O.K.N. *K*-SeT assay) and of VIM (RESIST-4 O.K.N.V. *K*-SeT assay) were developed (Pitout Johann et al., 2019). NG-Biotech Laboratories (France) has developed tests for detection of resistance to carbapenems (NG-Test CARBA 5: KPC, OXA-48-like, VIM, IMP, and NDM), third gen cephalosporins (NG-Test CTX-M MULTI: CTX-M) and colistin (NG-Test MCR-1: *mcr*). These tests are highly specific, require little hands-on time and deliver results within 15 min after loading (Pitout Johann et al., 2019; Bianco et al., 2020; Potron et al., 2020). However, these technologies need pure isolates and could not process clinical samples. The immunoassay-based field effect enzymatic detection biosensor, on the other hand, allowed direct detection and AST typing of low concentration *E*. *coli* in blood (∼10 CFU/ml) in less than 3 h (Shi et al., 2018).

## Novel Therapeutic Approaches

Overview over alternatives to current antibiotics discussed in this review is provided in Table 2. As of December 2020, there were 13 antibiotic drugs in Phase II clinical trials and 13–in Phase III clinical trials (PEW, 2021). Most of these drugs are modifications or combinations of already existing antibiotic scaffolds, but some are from novel synthetic classes. Gepotidacin (GSK2140944, Glaxo SmithKlein, United Kingdom) is a first drug in a novel synthetic class of triazaacenaphthylene bacterial topoisomerase inhibitors, aimed at treatment of uncomplicated urinary tract infections and urogenital gonorrhea. Its phase II clinical study demonstrated eradication of *Neisseria gonorrhoeae* in 95% of participants, and the drug has now entered the Phase III.

**TABLE 2:** Overview of alternatives to current antibiotics discussed in this review.

Class	Examples	Mode of action	Effective against	Clinical trial stage	Link
Novel antibiotics	Gepotidacin (GSK2140944, Glaxo SmithKlein, UK)	Synthetic drug, triazaacenaphtylene bacterial topoisomerase inhibitors	UTI and urogenital gonorrhea (*Neisseria gonorrhoeae*)	Phase III	https://www.gsk.com/en-gb/media/press-releases/gsk-starts-a-phase-iii-clinical-programme-for-a-potential-first-in-class-antibiotic-gepotidacin/
	Murepavadin (Polyphor AG, Switzerland)	Synthetic peptidomimetic drug, targets bacteria outer membrane protein	Cystic fybrosis (*Pseudomonas aeruginosa*)	Phase I (after discontinued Phase III for intravenous formulation)	https://www.polyphor.com/pol7080/
Antimicrobial resistance inhibitors	Beta-lactamase inhibitors	Inhibit serine beta-lactamase enzymes	ESKAPE pathogens	Approved	Ma et al. (2020)
	Efflux pump inhibitors	Prevent removal of antibiotic from the bacterial cell	ESKAPE pathogens	Preclinical tests	Sharma et al. (2019)
Bacteriocins	Nisin	Generates pores in the cell membrane	ESKAPE pathogens; *C*. *difficile*	Preclinical tests finished	Dijksteel et al. (2021) and Ma et al. (2020)
	Mersacidin	Inhibits cell wall biosynthesis	MRSA; VRE		
	Enterocin	Generates pores in the cell membrane	*Salmonella enterica*		
	NAI-107/NAI-108	Inhibits cell wall biosynthesis	MRSA, VRE, *Neisseria gonorrhoeae*		Brunati et al. (2018)
Bacteriophages	Biophage-PA	A cocktail of 6 bacteriophages (bacterial viruses) that infect bacteria, replicate in them, and then lyse them in order to infect other surrounding cells	Chronic otitis (*P*. *aeruginosa*)	Phase I/II completed	Wright et al. (2009)
	Exebacase (ContraFect, United States)	Bateriophage lysins	Bloodstream infections (*S*. *aureus* including MRSA)	Phase II completed	https://www.contrafect.com/pipeline/exebacase
	N-Rephasin (Intron Biotechnology, South Korea)			Phase II	https://intodeworld.com/the-worlds-first-clinical-trial-with-endolysin-based-bio-drug/
Nanoparticles	Silver, gold, copper, zinc, and iron NPs	Generate reactive oxygen species that disrupts membranes, inhibit cytochromes, destabilize ribosomes, damage DNA	*Salmonella typhi*, *S*. *aureus* (including MRSA), *E*. *coli*, *P*. *aeruginosa*, *S*. *enterica*, *K*. *pneumoniae*	Preclinical	Fatima et al. (2020)
Sequence-specific antimicrobials	Eligobiotics (Eligo Bioscience, France)	CRISPR-Cas based system; if DNA contains sites homologous to a guide RNA, the system becomes activated and DNA is fragmented by CRISPR-Cas nuclease	ESBL-*E*. *coli*; can be tailored to specific bacteria or AMR gene	Preclinical	https://anr.fr/Project-ANR-16-CE18-0021
Anti-virulence drugs	Essential oils of cinnamon, clove, thyme, marjoram	Inhibits quorum sensing mechanisms and thus prevents expression of virulence/pathogenic factors	ESKAPE pathogens	Preclinical	Alibi et al. (2020)
	Nanoparticles				Ali et al. (2020)
	Bacteriocins				Melian et al. (2019)
	Monoclonal antibodies		ESKAPE pathogens; *C*. *difficile*; *B*. *Anthracis*	Preclinical & clinical Phase I/II/III; FDA-approved	Zurawski and McLendon (2020)
Vaccination	–	Build up host immunity to a pathogen prior to a host encounter with that pathogen	*E*. *coli*, *S*. *aureus*, *P*. *aeruginosa*, and *K*. *pneumoniae*	Preclinical & clinical Phase I/II	Ma et al. (2020)

Murepavadin (Polyphor AG, Switzerland) is another example of a novel synthetic class antibiotic. It blocks lipopolysaccharide transport to the outer membrane of bacteria and was effective against treatment of *P*. *aeruginosa*, especially in cystic fibrosis patients. In 2018, the drug entered Phase III clinical trial for intravenous formulation, but it was discontinued by 2019 due to high incidence of acute kidney failure in patients (Smith, 2019). However, in December 2020 the company received another clinical trial authorization for an oral inhalation formulation of murepavadin against *P*. *aeruginosa* in cystic fibrosis patients and will start Phase I trial soon (Polyphor, 2021).

Discovery of novel antibiotics is an extremely time- and cost-consuming process. On average, it takes 10–15 years and around US $1.5 billion for an antibiotic to reach market after its first discovery (Towse et al., 2017). In addition, the limited and restricted use of an antibiotic after its approval, and inevitable resistance development toward the drug once it is in use, render big pharma companies reluctant to entering the business and many major players have left the stage (Plackett, 2020).

### Antimicrobial Resistance Inhibitors

Pathogens use a number of various mechanisms to withstand antibiotic treatment. They can modify cell wall proteins, produce enzymes for drug disruption, reduce cell influx by loss of porins, or induce drug wash out from the cell by activating efflux pumps. Apart from new antibiotics design, another strategy to overcome the resistance is to develop anti-resistance drugs which inactivate these mechanisms.

In 1970s, clavulanic acid–the first beta-lactamase inhibitor was discovered (Drawz and Bonomo, 2010). Beta-lactamase inhibitors disrupt beta-lactamases produced by resistant strains, which thus become susceptible to the drug. The most recent example of beta-lactam/beta-lactamase inhibitor approved for clinical use by FDA is imipenem/relebactam and meropenem/vaboractam for treatment of MDR ESKAPE pathogens. Other beta-lactam/beta-lactamase inhibitor combinations are undergoing preclinical and clinical trials now (Zhanel et al., 2018).

Efflux pump inhibitors prevent removal of antibiotic from cytoplasm. They have two modes of action, through energy dissipation or by blocking efflux pumps via direct binding (Sharma et al., 2019). These inhibitors exhibit promising activity in preclinical tests, but none of them have entered clinical trials yet (Ma et al., 2020). Efflux pumps have broad functional similarity across the tree of life, which brings additional challenge to finding molecules that would be specific for prokaryotes and would not have toxic effect on a host (Sharma et al., 2019). No commercially available efflux pump inhibitor drugs are available yet (AlMatar et al., 2021). However, nilotinib, an approved anti-cancer drug used to treat leukemia, has a potent activity against NorA efflux pump in *S*. *aureus* (Zimmermann et al., 2019).

### Bacteriocins

Nearly all bacteria produce antimicrobial peptides as a protection against other closely related species while staying immune to these peptides themselves (Yang et al., 2014). These peptides can be ribosomal or non-ribosomal. An example of non-ribosomal peptide is colistin, a last-resort antibiotic used against carbapenem-resistant pathogens. Colistin is produced in *Paenibacillus polymyxa* by three non-ribosomal peptide synthetases (Tambadou et al., 2015). Bacteriocins, on the other hand, are produced by ribosomes and do not require multiple enzymatic complexes (Yang et al., 2014). They have narrow to broad activity spectrum, need lower concentration to reach bactericidal effect compared to antibiotics, and do not exert toxic effect on human body (Negash and Tsehai, 2020). Having been widely used in food preservation for decades (And and Hoover, 2003), they also have a wide potential for treatment of infectious diseases. For example, nisin (produced by *Lactococcus lactis*) was found to be effective for treatment of MRSA skin infections; mersacidin (produced by *Bacillus* spp.) has inhibitory effect on MRSA and VRE; and enterocin (produced by *Enterococcus* spp.) exerts antimicrobial effect against *Salmonella enterica* (Ng et al., 2020).

Preclinical trials for three bacteriocins have been finished per date (Ma et al., 2020). NAI-107 bacteriocin and its derivative NAI-108 (bromine incorporated) have proved successful for treatment of MRSA, VRE, *Neisseria gonorrhoeae* even on non-dividing cells (Brunati et al., 2018). Its ability to kill persistent cells and destroy biofilms is of vital importance for treatment of infections caused by biofilm-forming bacteria, such as MRSA, or *P*. *aeruginosa*, since cells that are located in deep biofilm layers are metabolically inactive and do not respond to antibiotic treatment (Cascioferro et al., 2021).

Their incorporation with antibiotics may also help increase activity of existing antibiotics against pathogenic strains. For example, NAI-107/-108 exhibited synergistic effect against *A*. *baumanii*, *K*. *pneumoniae*, *E*. *coli*, and *P*. *aeruginosa*, when used with sub-inhibitory concentrations of polymyxins (Brunati et al., 2018). This potentially enables reduction of medication dosage decreasing the severity of polymyxin toxic effect on the host.

Large-scale production of natural bacteriocins might be a limiting factor for their pharmaceutical use, and here synthetic or bioengineered bacteriocins gain an advantage.

### Bacteriophages

Bacteriophages are bacteria-specific viruses and they have two types of replication. Lytic phages replicate in a host cytoplasm right after host cell infection, and then lyse the cell to infect other surrounding hosts (Guo et al., 2020). Temperate phages, on the other hand, replicate along with bacteria DNA after the host infection (lysogenic cycle) and remain dormant until the conditions for a lytic cycle are met. From a clinical perspective, lytic bacteriophages are a valuable asset in a battle against resistant bacteria (Forde and Hill, 2018). Phage therapy has several advantages over antibiotics. Phages are highly specific for a given species or even bacterial strain, they constantly coevolve with bacteria pathogens and since they only replicate inside the host bacteria cell, they are self-regulating (Domingo-Calap et al., 2016). Phage therapy was successfully used in Phase I/II clinical trial to treat 24 patients for chronic otitis caused by antibiotic resistant *P*. *aeruginosa* (Wright et al., 2009). Several studies report eradication of chronic prosthetic joint infections caused by MRSA, methicillin-sensitive *S*. *aureus* and *P*. *aeruginosa* after adjuvant use of bacteriophages with antibiotics (Ferry et al., 2018; Tkhilaishvili et al., 2019; Doub et al., 2020). Intra-nasal phage therapy caused *P*. *aeruginosa* load clearance in acute lung infection murine models (Chang et al., 2018).

Unfortunately, no regulations on phage therapy currently exist and all interventions are performed under the Helsinki Convention, i.e., only when all other treatment option ran out (Bassetti et al., 2017). Secondly, our immune system efficiently destroys bacteriophages, limiting use of intravenous formulations (Van Belleghem et al., 2018).

The other approach is to use not phages themselves, but lysins, phage bacteriolytic enzymes. Exebacase (lysin CF-301, ContraFect, United States) and N-Rephasin^®^ (SAL200 lysin, Intron Biotechnology, South Korea) target *S*. *aureus* biofilm and planktonic cells (including MRSA) and will be used for treatment of bloodstream infections. Exebacase successfully completed Phase II clinical trial for treatment and will soon start with Phase III trial (ContraFect, 2021). N-Rephasin^®^ is now in Phase II clinical trial (ClinicalTrials.gov, 2021).

### Nanoparticles

Metal ions (silver, zinc, copper, gold, and iron, etc.) have been long recognized for their antimicrobial activity. For example, silver has been used in ancient Greece, Egypt, and Roman Empire to preserve food and water and to treat wounds and ulcers (Alexander, 2009). Metal NPs mainly act through generation of reactive oxygen species that disrupt a wide range of metabolic processes including disruption of the membrane, inhibition of cytochromes, destabilization of ribosomes and DNA damage (Lee et al., 2019). Metal NPs prevent growth of such pathogens as *Salmonella typhi*, *S*. *aureus* (including MRSA), *E*. *coli*, *P*. *aeruginosa*, *S*. *enterica*, and *K*. *pneumoniae* (Fatima et al., 2020).

Nanoparticles conjugated with antibiotics have also demonstrated synergistic effect against MDR bacteria. For example, silver NPs in combination with vancomycin against *P*. *aeruginosa* resulted in fourfold increased MIC zone diameter, gold NPs coated with vancomycin enhanced its activity against VRE, zinc NPs with beta-lactams proved efficient against ESBL producing *K*. *pneumoniae*, *P*. *aeruginosa*, and *E*. *coli* causing urinary tract infections (Muzammil et al., 2018).

We should note though that bacteria can develop resistance toward NPs. For example, *E*. *coli* and *P*. *aeruginosa* can resist silver NPs through increased production of flagellin–a protein that triggers aggregation of NPs, without any genetic modification required (Panacek et al., 2018). However, changing the structure may help overcome this resistance. For example, silver-resistant *Salmonella* strains exhibited susceptibility to silver nanoring structured NPs (Gonzalez-Fernandez et al., 2020).

### Sequence-Specific Antimicrobials

Eligo Bioscience (France) is currently developing CRISPR-Cas based antimicrobials called eligobiotics (EligoBioscience, 2021). The sequence-specific antimicrobials technology (SSAM) is based on gene therapy, where exogenous CRISPR-Cas nuclease gene and its guide RNA will be delivered to the infection site. If bacterial DNA contains sequences homologous to the guide RNA, the system will be activated and bacterial DNA will be fragmented by a nuclease thus destroying a bacterial cell. Other bacteria will remain intact, allowing for targeted elimination of a pathogen (Bikard et al., 2014). The system can be tailored for a given strain/species or resistance genes.

### Anti-virulence Drugs

The strategy behind developing anti-virulence drugs is not to kill/inhibit growth of pathogenic bacteria, but to prevent them from exerting their pathogenicity. This can be achieved through interfering with quorum sensing (Bassetti et al., 2017). Quorum sensing regulates cellular processes though a production of self-inducing signal molecules that are secreted into environment. While bacteria load is small, these processes are kept silent to avoid detection by a host immune system or other bacteria (Zhao X. et al., 2020). Once the population density reaches a certain threshold, concentration of these signal molecules increases and bacteria start expressing genes for a better adaptation. Commonly these genes encode virulence factors, toxin production, and biofilm formation, etc. (Zhao X. et al., 2020). If bacteria become “deaf” to quorum sensing signals, on the other hand, they will not activate these mechanisms even when bacteria concentration is higher than a threshold. For example, degradation of a self-inducing molecule in *P*. *aeruginosa* in presence of azithromycin prevents biofilm formation, inhibits virulence factors expression and reduces growth rate by up to 90% at three sub-MICs (Cui et al., 2020). Essential oils of cinnamon, clove, thyme and marjoram exhibited high anti-biofilm, antioxidant and anti-quorum sensing activities against clinical isolates of *E*. *coli*, *K*. *pneumoniae*, *A*. *baumanii*, *S*. *aureus*, and *P*. *aeruginosa* (Alibi et al., 2020). Nanoparticles and bacteriocins also act as quorum sensing inhibitors (Melian et al., 2019; Ali et al., 2020). However, bacteria can develop resistance toward quorum sensing inhibitors and more research is needed before this strategy becomes widely used (Kalia et al., 2014). Human monoclonal antibodies (mAb) also can be used as anti-virulence drugs with several advantages over the common antibiotics. MAbs are highly selective and do not affect non-target microbiota, they provide rapid passive immunity against the pathogen and facilitate its immune-mediated clearance, and they do not exert toxic effect on humans (Martin-Galiano and McConnell, 2019). Currently, three mAbs against bacterial pathogens (raxibacumab and obiltoxaximab for inhalational anthrax and bezlotoxumab for recurrent *Clostridioides* difficile infection) are approved by FDA and 14 mAbs targeting various ESKAPE pathogens are under development in preclinical and clinical trials (Zurawski and McLendon, 2020). One of them, Salvecin^®^ (Aridis Pharmaceuticals, United States), targeting alpha-toxin of *S*. *aureus* (both MRSA and MSSA), is in Phase 3 trial.

### Vaccination

Vaccination is a prophylactic measure to build up host immunity against a pathogen prior to a host encounter with that pathogen. Previously it was rather difficult to develop effective vaccines against MDR bacteria, however, with a rise of reverse vaccinology this became a possibility (Rosini et al., 2020). Vaccines against resistant *E*. *coli*, *S*. *aureus*, *P*. *aeruginosa*, and *K*. *pneumoniae* are under development in preclinical and clinical trials (Ma et al., 2020).

In addition to direct vaccination against pathogens, vaccines against closely related species can also contribute to lowering risk of AMR spread. For example, massive vaccination against meningococcus B in Cuba, New Zealand and Canada led to an unexpected reduction in gonorrhea (Azze, 2019; Petousis-Harris and Radcliff, 2019). *N*. *meningitidis* and *N*. *gonorrhoeae* are 80–90% identical on genomic level, and thus probably share same antigens that antibodies developed under vaccination target. Vaccination against viruses also contributes to lowering antibiotics burden. For example, influenza vaccines prevent incidence of secondary bacterial infections in patients infected by influenza virus and thus reduce the need for antibiotic treatment (Micoli et al., 2021).

## Perspective

The COVID-19 pandemic has most likely worsened the already dire problem of AMR spread. Secondary bacterial infection is a common complication in viral pneumonia patients (Huttner et al., 2020). Therefore, from the start of the pandemic, doctors treating COVID-19 patients have been prone to prescribe antibiotics empirically on admission to hospital (Abelenda-Alonso et al., 2020). As such, up to 56–92% of hospitalized COVID-19 patients were given antibiotics throughout the course of treatment, although only around 6–15% of them actually suffered bacterial co-infection (Li et al., 2020; Vaughn et al., 2020; Martinez-Guerra et al., 2021). A retrospective study from the Wuhan Union Hospital revealed that the majority of patients with secondary bacterial infections had acquired antimicrobial resistant strains (carbapenem-resistant *K*. *pneumoniae*, *A*. *baumannii*; methicillin-resistant *S*. *aureus*) (Li et al., 2020).

Antimicrobial resistance is a complex problem that requires a multifaceted approach for its tackling. Advances in diagnostics will contribute to precise and fast identification of infectious agent, allowing doctors to conduct targeted infection treatment at the spot. At the same time, new approaches for infection therapy will facilitate effective treatment of MDR pathogens and ensure multifaceted strategies for killing of “stubborn” bugs with number of mechanisms to withstand antibiotics. Vaccination will reduce antibiotics use and ultimately prevent development of novel resistance mechanisms. Moreover, enhancement of hospital disinfection and wastewater treatment protocols and optimized hygiene routines for patients and healthcare workers will contribute to elimination of persistent pathogens in hospital environment minimizing the risk of MDR pathogen outbreaks and of their escape into environment. Also, some antibiotics lack breakpoints for some pathogens, which also sets limitations that need to be addressed in the future. However, with all the advancements, scientists and pharma alone will not be able to change the game. Governments need to step in to educate the public and encourage big pharma to re-enter the antimicrobial development. Public awareness programs, such as the World Antimicrobial Awareness Week organized by the WHO, need to be more pronounced and reach more people, and antibiotics use must be strictly regulated in all countries. We believe that if we all stand together and use our assets properly, we have a fair chance to reverse the trend and win the game against MDR bacteria.

## Author Contributions

EA, VS, and GS conceived the idea and summarized figure and tables. EA drafted the manuscript. All authors have edited the manuscript and agreed on its final version.

## Conflict of Interest

The authors declare that the research was conducted in the absence of any commercial or financial relationships that could be construed as a potential conflict of interest.
